# Long-fragment DNA as a potential marker for stool-based detection of colorectal cancer

**DOI:** 10.3892/ol.2014.2632

**Published:** 2014-10-24

**Authors:** YIBO ZHANG, YUTAKA SUEHIRO, YOSHITARO SHINDO, KOUHEI SAKAI, SHOICHI HAZAMA, SHINGO HIGAKI, ISAO SAKAIDA, MASAAKI OKA, TAKAHIRO YAMASAKI

**Affiliations:** 1 Department of Oncology and Laboratory Medicine, Yamaguchi University Graduate School of Medicine, Ube, Yamaguchi 755-8505, Japan; 2 Department of Digestive Surgery and Surgical Oncology (Surgery II), Yamaguchi University Graduate School of Medicine, Ube, Yamaguchi 755-8505, Japan; 3 Department of Gastroenterology and Hepatology, Yamaguchi University Graduate School of Medicine, Ube, Yamaguchi 755-8505, Japan

**Keywords:** colorectal cancer, stool DNA test

## Abstract

Neoplastic cells that are exfoliated from the colorectal epithelium exhibit dysfunctional apoptotic mechanisms, and thus it is possible to identify high-molecular-weight DNA fragments (long DNA) in feces. In the present study, the sensitivity and specificity of fecal-based long DNA assays were evaluated for the detection of colorectal cancer (CRC). Feces were collected from 54 healthy volunteers and 130 patients with CRC prior to surgical treatment. The presence of long DNA of the adenomatosis polyposis coli, Kirsten rat sarcoma viral oncogene homolog (*KRAS)*, B-raf proto-oncogene, serine/threonine kinase and *p53* genes was assessed by polymerase chain reaction followed by electrophoresis. The identification of long DNA in feces was found to exhibit a sensitivity of 56.2% and specificity of 96.3% for CRC detection. In addition, long DNA was identified in the feces of 58/90 (64.4%) patients with distal CRC and 15/40 (37.5%) patients with proximal CRC. This study indicates the potential of the fecal long DNA assay as a non-invasive and easily performed method for the detection of individuals with CRC.

## Introduction

Colorectal cancer (CRC) is the fourth leading cause of cancer-associated mortality in males and the third leading cause in females worldwide (1). It is estimated that 1,200,000 new cases of CRC and 608,700 CRC-associated mortalities occurred in 2008 (1). As >95% of patients with CRC would benefit from curative surgery if diagnosed at an earlier or precancerous stage (2), it is important to develop highly sensitive and specific assays to detect CRC earlier, which are non-invasive, inexpensive and easy to perform. To date, a large number of methods have been developed for the early detection of CRC, including endoscopic examinations and blood- and stool-based tests (2). Colonoscopy is regarded as the gold standard procedure due to its capacity to identify and remove precancerous and cancerous lesions. However, due to its invasiveness, patient compliance with this procedure is poor. Although various blood tests using protein, cytological, microRNA and DNA markers have been investigated, the majority are not suitable for clinical application (3). The main approach used for CRC screening worldwide is the fecal occult blood test (FOBT) (3). However, the sensitivity of the FOBT for CRC diagnosis is only 26% for the detection of carcinoma and 12% for advanced adenoma (4). Furthermore, FOBT presents the risk of false-positive results in patients with hemorrhoids, ulcers and inflammatory bowel disease (5–7). To avoid the disadvantages of the FOBT, more sensitive and specific screening methods are required.

A variety of fecal molecular markers, including mutations of oncogenes and tumor suppressor genes, microsatellite instability and microRNA and DNA methylation may increase the sensitivity of CRC screening (8–14). In particular, the fecal long DNA assay appears to present a valid and reliable method for the detection of CRC (15–19). Long DNA is DNA from cancerous or precancerous cells, which is shed from dysplastic mucosa, and thus maintains a longer-fragment DNA form due to its resistance to the apoptotic process (20). By contrast, during the apoptotic process, DNA is cleaved and a 200 bp DNA fragment is yielded (21). Although various advanced technologies have been applied to analyze long DNA markers in stools, such time-consuming and expensive methods are not appropriate for the screening of CRC. In the current study, a long DNA assay was performed using polymerase chain reaction (PCR) and electrophoresis and the combination of different long DNA markers was found to increase the diagnostic performance of this method for CRC detection.

## Materials and methods

### Clinical samples

Stool samples were obtained from 54 healthy volunteers and 130 patients with CRC prior to surgical treatment. The clinicopathological features of the patients are shown in Table I. The mean age of the patients was 68.1 years (range, 37–94 years; 84 males and 46 females). A total of eight patients exhibited stage 0, 47 exhibited stage I, 24 exhibited stage II, 33 exhibited stage III and 18 exhibited stage IV CRC according to the Japanese Society for Cancer of the Colon and Rectum staging system (22). A total of 40 patients had right-sided CRC (proximal CRC) involving the cecum, ascending colon and transverse colon and 90 patients had left-sided CRC (distal CRC), involving the descending colon, sigmoid colon and rectum. The study protocol was approved by the ethics committee of Yamaguchi University Graduate School of Medicine (Ube, Japan) and informed consent was obtained from each patient and the volunteers.

### DNA extraction

Fecal specimens were obtained from Yamaguchi University Hospital (Ube, Japan) and stored at −20°C prior to DNA extraction. Fecal samples were thawed and 100–200 mg of fecal samples were used for DNA extraction. The DNA was extracted using the QIAamp DNA Stool Mini kit (Qiagen, Hilden, Germany) according to the manufacturer’s instructions. Extracted DNA was diluted to a final concentration of 20 ng/μl.

### PCR

PCR was performed for four different genes, including adenomatosis polyposis coli (APC), B-raf proto-oncogene, serine/threonine kinase (BRAF), Kirsten rat sarcoma viral oncogene homolog (KRAS) and p53 for the stool long DNA tests. The PCR reaction was performed using 40 ng of DNA, 1X Buffer II (Applied Biosystems Life Technologies, Foster City, CA, USA), 1.5 mM MgCl_2_, 0.8 mM dNTPs, 1 μM of each primer, 3% dimethyl sulfoxide, and 0.25 U AmpliTaq Gold DNA polymerase (Applied Biosystems Life Technologies) in a total volume of 10 μl. The primers used are shown in Table II. Cycling conditions included preheating at 95°C for 7 min followed by 45 cycles of denaturation at 95°C for 45 sec, annealing at 60°C for 30 sec and extension at 72°C for 1 min. β-actin (Fasmac, Kanagawa, Japan) was amplified as an internal control. The PCR products were then electrophoresed on 2% agarose gel and visualized by ethidium bromide staining.

### Statistical analysis

Statistical analysis was performed using GraphPad Prism version 6.0 and GraphPad InStat version 3.0 statistical software (GraphPad Software, La Jolla, CA, USA). To compare variables, Pearson’s χ^2^ and Fisher’s tests were used and P<0.05 was considered to indicate a statistically significant difference.

## Results

### Long DNA

**Figure 1:**
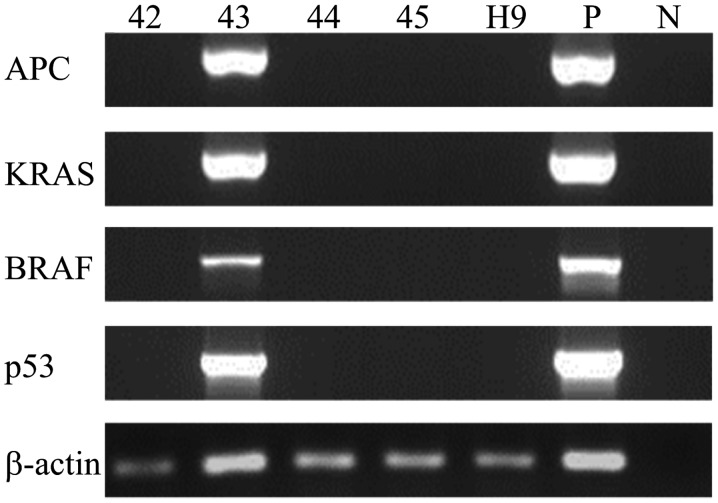
Gel electrophoresis analysis of the long DNA assay. PCR products of APC, KRAS, BRAF and p53 PCR were observed in case 43 but not in other cases. β-actin was amplified as an internal control. P, positive control; N, negative control (water); PCR, polymerase chain reaction; APC; adenomatosis polyposis coli; KRAS; Kirsten rat sarcoma viral oncogene homolog; BRAF; B-raf proto-oncogene, serine/threonine kinase.

A representative result obtained from the long DNA assays following gel electrophoresis is shown in Fig. 1. The frequency of long DNA of the four genes was significantly higher in the feces of the CRC patients than that of the controls (Table III). *APC* long DNA was identified in the feces of 60/130 (46.2%) patients with CRC and 1/54 (1.9%) of the controls (P<0.0001). *KRAS* long DNA was identified in 50/130 (38.5%) CRC patients and 1/54 (1.9%) controls (P<0.0001). *BRAF* long DNA was identified in 51/130 (39.2%) CRC patients and 0/54 (0.0%) controls (p<0.0001). *p53* long DNA was identified in 44/130 (33.8%) CRC patients and 0/54 (0.0%) controls (P<0.0001). Furthermore, a combination of the four genes exhibited a sensitivity of 56.2% and a specificity of 96.3% for the detection of CRC (P<0.0001).

### Association between long DNA and clinicopathological parameters

The association between long DNA and clinicopathological features is shown in Table III. No significant difference was identified between long DNA and gender or tumor stage. The frequency of long DNA was significantly higher in the feces of patients with distal CRC when compared with that in the feces of patients with proximal CRC. *APC* long DNA was identified in 49/90 (54.4%) patients with distal CRC and 11/40 (27.5%) patients with proximal CRC (P=0.0072). *KRAS* long DNA was identified in 43/90 (47.8%) patients with distal CRC and 7/40 (17.5%) patients with proximal CRC (p=0.0010). *BRAF* long DNA was identified in 43/90 (47.8%) patients with distal CRC and 8/40 (20%) patients with proximal CRC (P=0.0033). *p53* long DNA was identified in 37/90 (41.1%) patients with distal CRC and in 7/40 (17.5%) patients with proximal CRC (P=0.0093). Furthermore, one or more of the four long DNAs was identified in 58/90 (64.4%) patients with distal CRC and 15/40 (37.5%) patients with proximal CRC (P=0.0069).

## Discussion

In the current study, the efficacy of fecal long DNA as a potential marker for CRC screening was demonstrated. Longer template DNA is an epigenetic phenomenon that is consistent with the known abrogation of apoptosis that occurs with CRC (20,23). It is hypothesized that nonapoptotic cells are exfoliated from neoplasms (24), however, by contrast, colonocyte shedding from normal mucosa is relatively sparse and sloughed cells appear to be largely apoptotic (20,24). In addition, apoptosis of normal cells rapidly occurs following detachment from their basement membrane (25). As the cleavage of DNA by endonucleases into fragments of 180–200 bp (21,26) is an attribute of apoptosis, it follows that human DNA in normal stools would exist primarily in fragmented forms. However, the stools of CRC patients are also expected to contain nonapoptotic long DNA. In the current study, a long DNA assay based on PCR of an 800 bp length amplicon of *APC*, *KRAS*, *BRAF* and *p53* genes was performed for CRC screening. These genes were selected based on the study by Kalimutho *et al* (14), however, the primers were redesigned to yield a shorter amplicon size than that used in the previous study (1015–1340 bp) (14). The sensitivity of the long DNA assay using a combination of the four genes was increased when compared with that of each single gene alone. In addition, the sensitivity was higher in distal CRC than in proximal CRC. The effective detection of distal colon neoplasms is critical, as >70% of CRCs occur in the distal colon (27).

Alu-based PCR (Alu-PCR) and quantitative-denaturing high performance liquid chromatography (QdHPLC)-PCR methods have also been used to detect fecal long DNA for CRC screening (14,28). The sensitivity of Alu-PCR was marginally lower (44.0%) and its specificity was marginally higher (100.0%) when compared with the results of the present study (sensitivity, 56.2%; specificity, 96.3%) (P<0.0001) (28). QdHPLC-PCR, which evaluates PCR amplicons of the same four genes used in the present study (APC, KRAS, p53 and BRAF) (14), exhibited a higher sensitivity (78.6%) and a marginally lower specificity (91.6%) for CRC screening when compared with the results of the present study (sensitivity, 56%; specificity, 96%) (P<0.0001). The predominant disadvantage of QdHPLC-PCR is the requirement for expensive equipment, which results in costly tests. By contrast, the method used in this study requires only a thermal cycler and an electrophoresis device, thus allowing the detection of the long DNA with minimal expense.

In conclusion, the current study indicates that the detection of fecal long DNA by PCR and electrophoresis is a valid, feasible and inexpensive method for the identification of patients with CRC, particularly in patients with distal CRC. However, as sensitivities of 56.2% for the detection of CRC and 64.4% for the detection of distal CRC by the long DNA assay alone require improvement, further optimization of the long DNA assay in combination with other methods including FOBT and other stool DNA tests are needed for future clinical use. In addition, further studies of long DNA assays for the identification of patients with advanced adenoma are also required.

## Figures and Tables

**Table I:** Summary of the clinical patient data (n=130).

Clinical Parameters	n
Gender	
Male	84
Female	46
Mean age, years (range)	68.1 (37–94)
Stage	
0	8
I	47
II	24
III	33
IV	18
Tumor site	
Ascending colon	27
Transverse colon	13
Descending colon	9
Sigmoid colon	43
Rectum	38

Staging was according to the Japanese Society for Cancer of the Colon and Rectum (22).

**Table II:** Primer sequences used for polymerase chain reaction.

Gene	Primer sequence	Primer T_m_,°C	Bp, n
APC	Forward: 5′-TATGCGTGTCAACTGCCATC-3′	63.2	800
	Reverse: 5′-CTCTGTTTTGGCGACGTCTA-3′	63.8	
KRAS	Forward: 5′-AGACTTGGGAGTCTTCGATCC-3′	63.3	800
	Reverse: 5′-CTTACTGGCACCTAGGTTAG-3′	64.0	
BRAF	Forward: 5′-CCATAGCATGAAGGCAGGTT-3′	63.8	800
	Reverse: 5′-CGTGTCGGTTTCAATCACGT-3′	63.2	
p53	Forward: 5′-TCACCATCGCTATCTGAGCA-3′	64.7	800
	Reverse: 5′-AAACCCTGTCCTCAGTCTCTAG-3′	63.8	
β-actin	Forward: 5′-TCATCTTCTCGCGGTTGGC-3′	68.8	103
	Reverse: 5′-CGGTTGGCGCTCTTCTACT-3′	66.9	

APC; adenomatosis polyposis coli; KRAS; Kirsten rat sarcoma viral oncogene homolog; BRAF; B-raf proto-oncogene, serine/threonine kinase; T_m_, melting temperature; Bp, base pairs.

**Table III:** Comparison of long DNA with clinicopathological parameters.

Clinicopathological parameters (n)	APC, n (%)	KRAS, n (%)	BRAF, n (%)	p53, n (%)	Biomarker panel, n (%)
Patients					
CRC (130)	60 (46.2)	50 (38.5)	51 (39.2)	44 (33.8)	73 (56.2)
Control (54)	1 (1.9)	1 (1.9)	0 (0)	0 (0)	2 (3.7)
P value	<0.0001	<0.0001	<0.0001	<0.0001	<0.0001
Gender					
Male (84)	42 (50)	33 (39.3)	35 (41.7)	30 (35.7)	50 (59.5)
Female (46)	18 (39.1)	17 (37.0)	16 (34.8)	14 (30.4)	23 (50.0)
P-value	NS	NS	NS	NS	NS
Tumor site					
Proximal (40)	11 (27.5)	7 (17.5)	8 (20.0)	7 (17.5)	15 (37.5)
Distal (90)	49 (54.4)	43 (47.8)	43 (47.8)	37 (41.1)	58 (64.4)
P-value	0.0072	0.001	0.0033	0.0093	0.0069
TNM stage					
0 (8)	3 (37.5)	2 (25.0)	2 (25.0)	2 (25.0)	3 (37.5)
I (47)	23 (48.9)	21 (44.7)	21 (44.7)	16 (34.0)	29 (61.7)
II (24)	10 (41.7)	9 (37.5)	11 (48.5)	10 (41.7)	12 (50.0)
III (33)	13 (39.4)	11 (33.3)	12 (36.4)	9 (27.3)	16 (48.5)
IV (18)	11 (61.1)	7 (38.9)	5 (27.8)	7 (38.9)	13 (72.2)
P-value	NS	NS	NS	NS	NS

Biomarker panel means positive long DNA for at least one marker. NS, not significant; TNM, tumor node metastasis; CRC, colorectal cancer; APC; adenomatosis polyposis coli; KRAS; Kirsten rat sarcoma viral oncogene homolog; BRAF; B-raf proto-oncogene, serine/threonine kinase.
